# CO_2_-dependent carbon isotope fractionation in dinoflagellates relates to their inorganic carbon fluxes

**DOI:** 10.1016/j.jembe.2016.04.001

**Published:** 2016-08

**Authors:** Mirja Hoins, Tim Eberlein, Dedmer B. Van de Waal, Appy Sluijs, Gert-Jan Reichart, Björn Rost

**Affiliations:** a Department of Earth Sciences, Faculty of Geosciences, Utrecht University, Budapestlaan 4, 3584 CD Utrecht, The Netherlands; b Marine Biogeosciences, Alfred Wegener Institute, Helmholtz Centre for Polar- and Marine Research, Am Handelshafen 12, 27570 Bremerhaven, Germany; c Department of Aquatic Ecology, Netherlands Institute of Ecology (NIOO-KNAW), Droevendaalsesteeg 10, 6708 PB Wageningen, The Netherlands; d Royal Netherlands Institute for Sea Research (NIOZ), Landsdiep 4, 1797 SZ ‘t Horntje, Texel, The Netherlands

**Keywords:** C_i_, inorganic carbon, CCM, CO_2_-concentrating mechanism, Chl-*a*, Chlorophyll-*a*, ε_p_, carbon isotope fractionation, ε_p-meas_, measured carbon isotope fractionation, ε_p-mod_, modeled carbon isotope fractionation, ε_s_, equilibrium fractionation between CO_2_ and HCO_3_^−^, ε_f_, kinetic fractionation associated with the CO_2_ fixation of RubisCO, L_CO2_, ratio of CO_2_ efflux relative to total C_i_ uptake, DIC, dissolved inorganic carbon, HCO_3_^−^,  bicarbonate, R_HCO3_, ratio of HCO_3_^−^ to total C_i_ uptake, RubisCO, ribulose-1,5-bisphosphate Carboxylase/Oxygenase, CA, carbonic anhydrase, TA, total alkalinity, CCM, CO_2_ uptake, HCO_3_^−^ uptake, Leakage

## Abstract

Carbon isotope fractionation (ε_p_) between the inorganic carbon source and organic matter has been proposed to be a function of *p*CO_2_. To understand the CO_2_-dependency of ε_p_ and species-specific differences therein, inorganic carbon fluxes in the four dinoflagellate species *Alexandrium fundyense, Scrippsiella trochoidea, Gonyaulax spinifera* and *Protoceratium reticulatum* have been measured by means of membrane-inlet mass spectrometry. In-vivo assays were carried out at different CO_2_ concentrations, representing a range of *p*CO_2_ from 180 to 1200 μatm. The relative bicarbonate contribution (i.e. the ratio of bicarbonate uptake to total inorganic carbon uptake) and leakage (i.e. the ratio of CO_2_ efflux to total inorganic carbon uptake) varied from 0.2 to 0.5 and 0.4 to 0.7, respectively, and differed significantly between species. These ratios were fed into a single-compartment model, and ε_p_ values were calculated and compared to carbon isotope fractionation measured under the same conditions. For all investigated species, modeled and measured ε_p_ values were comparable (*A. fundyense, S. trochoidea, P. reticulatum*) and/or showed similar trends with *p*CO_2_ (*A. fundyense, G. spinifera, P. reticulatum*). Offsets are attributed to biases in inorganic flux measurements, an overestimated fractionation factor for the CO_2_-fixing enzyme RubisCO, or the fact that intracellular inorganic carbon fluxes were not taken into account in the model. This study demonstrates that CO_2_-dependency in ε_p_ can largely be explained by the inorganic carbon fluxes of the individual dinoflagellates.

## Highlights

• To understand ^13^C fractionation in dinoflagellates, inorganic carbon fluxes were measured by MIMS and used for modeling.
• Changes in cellular carbon fluxes, i.e. HCO_3_^−^ contribution and CO_2_ leakage were CO_2_-dependent.
• These CO_2_-dependencies could largely explain the CO_2_-dependent fractionation patterns observed in the four tested species.

## 1 Introduction

During photosynthetic carbon fixation, the lighter carbon isotope ^12^C is preferred over the heavier carbon isotope ^13^C, thereby causing carbon isotope fractionation (ε_p_) between the inorganic carbon (C_i_) source and the organic carbon. Values for ε_p_ of marine phytoplankton have been shown to be CO_2_-sensitive (e.g. Degens et al., 1968), and thus were discussed to serve as a proxy for past CO_2_ concentrations (Jasper and Hayes, 1990, Pagani, 2014, Van de Waal et al., 2013, Hoins et al., 2015). Large species-specific differences in ε_p_ have been described, which are yet poorly understood (e.g. Hinga et al., 1994, Burkhardt et al., 1999). Moreover, irrespective of the phytoplankton species investigated, most of these studies have solely described the relationship between ε_p_ and CO_2_, and only few have investigated the underlying physiological processes. Such mechanistic understanding is, however, needed to identify the reasons of the CO_2_-dependency of ε_p_.

Carbon isotope fractionation of phytoplankton is primarily driven by the enzyme ribulose-1,5-bisphosphate Carboxylase/Oxygenase (RubisCO), which is responsible for the fixation of CO_2_ into organic compounds. The intrinsic fractionation associated with RubisCO (ε_f_) has been estimated to range between ~ 22 and 30‰ (e.g. Roeske and O'Leary, 1984, Guy et al., 1993, Scott et al., 2007), even though a recent study obtained values as low as 11‰ for the RubisCO of the coccolithophore *Emiliania huxleyi* (Boller et al., 2011). While RubisCO principally sets the upper limit of fractionation, other processes strongly determine the degree to which RubisCO can express its fractionation (Sharkey and Berry, 1985, Burkhardt et al., 1999, Rost et al., 2002). First, there is leakage, i.e. the amount of CO_2_ diffusing out of the cell in relation to C_i_ uptake. With higher leakage, the intracellular C_i_ pool is ‘refreshed’, thereby preventing accumulation of ^13^C and allowing RubisCO to approach its upper fractionation values. Second, the relative contribution of bicarbonate (HCO_3_^−^) to total C_i_ uptake plays a role, as HCO_3_^−^ is enriched in ^13^C by ~ 10‰ relative to CO_2_ (Mook et al., 1974). An increasing HCO_3_^−^ contribution thus lowers ε_p_. The enzyme carbonic anhydrase (CA), which accelerates the otherwise slow interconversion between CO_2_ and HCO_3_^−^, can also influence ε_p_ under certain conditions, e.g. by influencing leakage as well as the relative HCO_3_^−^ contribution. All these processes play a role in the CO_2_-concentrating mechanisms (CCMs) of phytoplankton. Assessing the mode of CCMs may therefore help to understand the reasons for CO_2_-dependent changes in ε_p_ and species-specific differences therein.

Dinoflagellates are cosmopolitan unicellular algae that occur in many different environments, including eutrophic coastal regions and oligotrophic open oceans. In this study, we investigated whether the CO_2_-dependency of ε_p_, which was found in the dinoflagellate species *Alexandrium fundyense, Gonyaulax spinifera*, *Protoceratium reticulatum* and *Scrippsiella trochoidea* (Burkhardt et al., 1999, Hoins et al., 2015), can be explained by changes in their C_i_ fluxes. Characteristics of CCMs in the tested species, including their CA activities and C_i_ fluxes, were measured by means of membrane-inlet mass spectrometry (MIMS). Results were fed into a single-compartment model that considers cellular leakage, the relative HCO_3_^−^ contribution as well as the carbon isotope fractionation of RubisCO (Sharkey and Berry, 1985, Burkhardt et al., 1999). The calculated carbon fractionation (ε_p-mod_) was then compared to the measured carbon fractionation (ε_p-meas_).

## 2 Material and methods

### 2.1 Incubations

Cultures of the dinoflagellate species *A. fundyense* (formerly *Alexandrium tamarense* strain Alex5; John et al., 2014), *S. trochoidea* (strain GeoB267; culture collection of the University of Bremen), *G. spinifera* (strain CCMP 409) and *P. reticulatum* (strain CCMP 1889) were grown in 0.2 μm filtered North Sea water (salinity 34), which was enriched with 100 μmol L^− 1^ nitrate and 6.25 μmol L^− 1^ phosphate. Metals and vitamins were added according to f/2 medium (Guillard and Ryther, 1962), except for FeCl_3_ (1.9 μmol L^− 1^), H_2_SeO_3_ (10 nmol L^− 1^) and NiCl_2_ (6.3 nmol L^− 1^) that were added according to K medium (Keller et al., 1987). Each of the strains was grown in 2.4 L air-tight borosilicate bottles at 15 °C and 250 ± 25 μmol photons m^− 2^ s^− 1^ at a 16:8 h light:dark cycle. Bottles were placed on roller tables in order to avoid sedimentation.

Dissolved CO_2_ concentrations ranged from ~ 5–50 μmol L^− 1^ and were reached by pre-aerating culture medium with air containing 180, 380, 800 and 1200 μatm *p*CO_2_. The carbonate chemistry was calculated based on pH and total alkalinity (TA), using the program CO2sys (Pierrot et al., 2006). pH values were measured using a WTW 3110 pH meter equipped with a SenTix 41 Plus pH electrode (WTW, Weilheim, Germany), which was calibrated prior to measurements to the National Bureau of Standards (NBS) scale. An automated TitroLine burette system (SI Analytics, Mainz, Germany) was used to determine TA. Dissolved inorganic carbon (DIC) was determined colorimetrically using a QuAAtro autoanalyser (Seal Analytical, Mequon, USA). For more details on the carbonate chemistry in the acclimations, please refer to Eberlein et al. (2014) for *A. fundyense* and *S. trochoidea* and to Hoins et al. (2015) for *G. spinifera* and *P. reticulatum.*

To determine ε_p_ values, the isotopic composition of the organic material was measured using an Automated Nitrogen Carbon Analyser mass spectrometer (ANCA-SL 20–20, SerCon Ltd., Crewe, UK), and the isotopic composition of the DIC in growth medium was measured using a GasBench-II coupled to a Thermo Delta-V advantage isotope ratio mass spectrometer (see Hoins et al., 2015 for details on isotope analysis). Prior to assays, cells were acclimated to the different CO_2_ concentrations for at least 7 generations (i.e. > 21 days). To prevent changes in the carbonate chemistry, i.e. keeping drawdown of DIC < 3%, incubations were terminated at low cell densities (< 400 cells mL^− 1^).

### 2.2 MIMS assays

A custom-made membrane-inlet mass spectrometer (MIMS; Isoprime, GV Instruments, Manchester, UK; see Rost et al., 2007 for details) was used to determine CA activities and C_i_ fluxes of *A. fundyense* and *S. trochoidea* acclimated to four different *p*CO_2_ (i.e. 180, 380, 800 and 1200 μatm; Eberlein et al., 2014), and of *G. spinifera* and *P. reticulatum* acclimated to a low and high *p*CO_2_ (i.e. 180 and 800 μatm). Assays were performed in an 8 mL temperature-controlled cuvette, equipped with a stirrer. Assay tests over ~ 1 h confirmed that conditions during the assay do not cause physiological stress (i.e. no decline in O_2_ production rates), and subsequent microscopic inspection did not reveal any visual effects on cell morphologies. Prior to the measurements, acclimated cells were concentrated using a 10 μm membrane filter (Millipore, Billerica, MA) by gentle vacuum filtration (< 200 mbar) and stepwise transferred into C_i_-free medium buffered with a 4-(2-hydroxylethyl)-1-piperazine-ethanesulfonic acid (50 mmol^− 1^ HEPES) solution at 15 ± 0.3 °C and a pH of 8.0 ± 0.1. Chlorophyll *a* (Chl-*a*) concentrations were determined fluorometrically by using a TD-700 Fluorometer (Turner Designs, Sunnyvale, CA, USA) and ranged between 0.15 and 1.70 μg mL^− 1^ during the assays.

To quantify activities of extracellular CA (eCA), the ^18^O depletion rate of doubly labeled ^13^C^18^O_2_ in seawater was determined by measuring the transient changes in ^13^C^18^O^18^O (m/z = 49), ^13^C^18^O^16^O (m/z = 47) and ^13^C^16^O^16^O (m/z = 45) in the dark, following the approach of Silvermann (1982). If cells possess eCA, exchange rates of ^18^O are accelerated relative to the spontaneous rate. To monitor the spontaneous rate, NaH^13^C^18^O_3_ label was injected to the cuvette, waiting until the m/z = 49 signal reached a steady-state decline. This rate was then compared to the steady-state decline after cells were added. Following Badger and Price (1989), eCA activity is expressed as percentage decrease in ^18^O-atom fraction upon the addition of cells, normalized to Chl-*a*. Consequently, 100 units (U) correspond to a doubling in the rate of interconversion between CO_2_ and HCO_3_^−^ per μg Chl-*a*.

Photosynthetic O_2_ and C_i_ fluxes were determined following Badger et al. (1994). Making use of the chemical disequilibrium, this approach estimates CO_2_ and HCO_3_^−^ fluxes during steady-state photosynthesis. It is based on the simultaneous measurements of O_2_ and CO_2_ concentrations during consecutive light and dark intervals with increasing amounts of DIC. Oxygen fluxes in the dark and light are converted into C_i_ fluxes by applying a respiratory quotient of 1.0 and a photosynthetic quotient of 1.1 (Burkhardt et al., 2001, Rost et al., 2003). The light intensity in the cuvette was adjusted to the acclimation conditions (i.e. 250 ± 25 μmol photons m^− 2^ s^− 1^). Net CO_2_ uptake was calculated from the steady-state decline in CO_2_ concentration at the end of the light period, corrected for the interconversion between CO_2_ and HCO_3_^−^. The uptake of HCO_3_^−^ was calculated by subtracting net CO_2_ uptake from net C_i_ uptake, and the CO_2_ efflux from the cells was estimated from the initial slope after turning off the light. Rate constants *k*_*1*_ and *k*_*2*_ were determined based on temperature, salinity and pH (Zeebe and Wolf-Gladrow, 2001, Schulz et al., 2006), yielding mean values of 0.9241 (± 0.0506) min^− 1^ and 0.0085 (± 0.0008) min^− 1^, respectively. To eliminate any eCA activity, a prerequisite to apply the rate constants, we added dextran-bound sulfonamide (DBS; 50 μmol L^− 1^) to the cuvette. For more details on the calculations, please refer to Badger et al. (1994) and Schulz et al. (2007).

### 2.3 Single-compartment model

To calculate ε_p-mod_, results for the relative HCO_3_^−^ contribution and leakage were fed into a single-compartment model after Sharkey and Berry (1985) and Burkhardt et al. (1999):

(1) $$\varepsilon_{p-\mathrm{mod}}=R_{\mathrm{HCO}3}\times \varepsilon_{s}+L_{\mathrm{CO}2}\times \varepsilon_{f}$$

where R_HCO3_ represents the ratio of HCO_3_^−^ to total C_i_ uptake, ε_s_ the equilibrium fractionation between CO_2_ and HCO_3_^−^ (− 10‰; Mook et al., 1974), L_CO2_ the ratio of CO_2_ efflux relative to total C_i_ uptake, and ε_f_ the kinetic fractionation associated with the CO_2_ fixation of RubisCO, which was here assumed to be 28‰ after Raven and Johnston (1991).

### 2.4 Statistical analysis

Shapiro–Wilk tests confirmed normality of the data. Significant differences between CO_2_ treatments were confirmed by a one-way ANOVA followed by post hoc comparison of the means using the Tukey HSD (α = 0.05; Table 1).

## 3 Results

### 3.1 CA activity

In *A. fundyense* and *P. reticulatum*, eCA activities were low with maximum activities of 156 U (μg Chl-*a*)^− 1^ and 44 U (μg Chl-*a*)^− 1^, respectively. In *S. trochoidea* and *G. spinifera*, eCA activities were comparably high with up to 1600 U (μg Chl-*a*)^− 1^ and 1100 U (μg Chl-*a*)^− 1^, respectively. In neither of the species, eCA activities were responding to changes in *p*CO_2_. Please note that for *G. spinifera* and *P. reticulatum* no statistics could be applied due to the lack of replication.

### 3.2 HCO_3_^−^ contribution and leakage

**Fig. 1:**
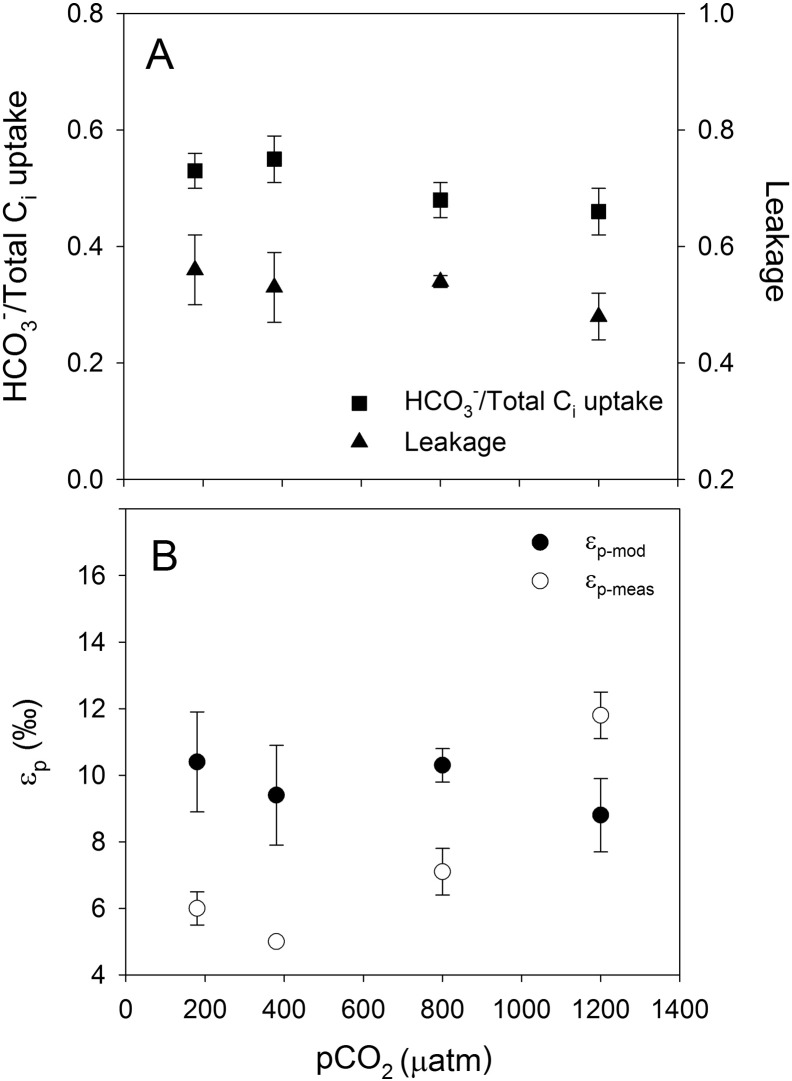
Relative HCO_3_^−^ contribution, leakage and ε_p-mod_ and ε_p-meas_ in *A. fundyense*. Each data point represents the mean ± standard deviation (n = 3).

**Fig. 2:**
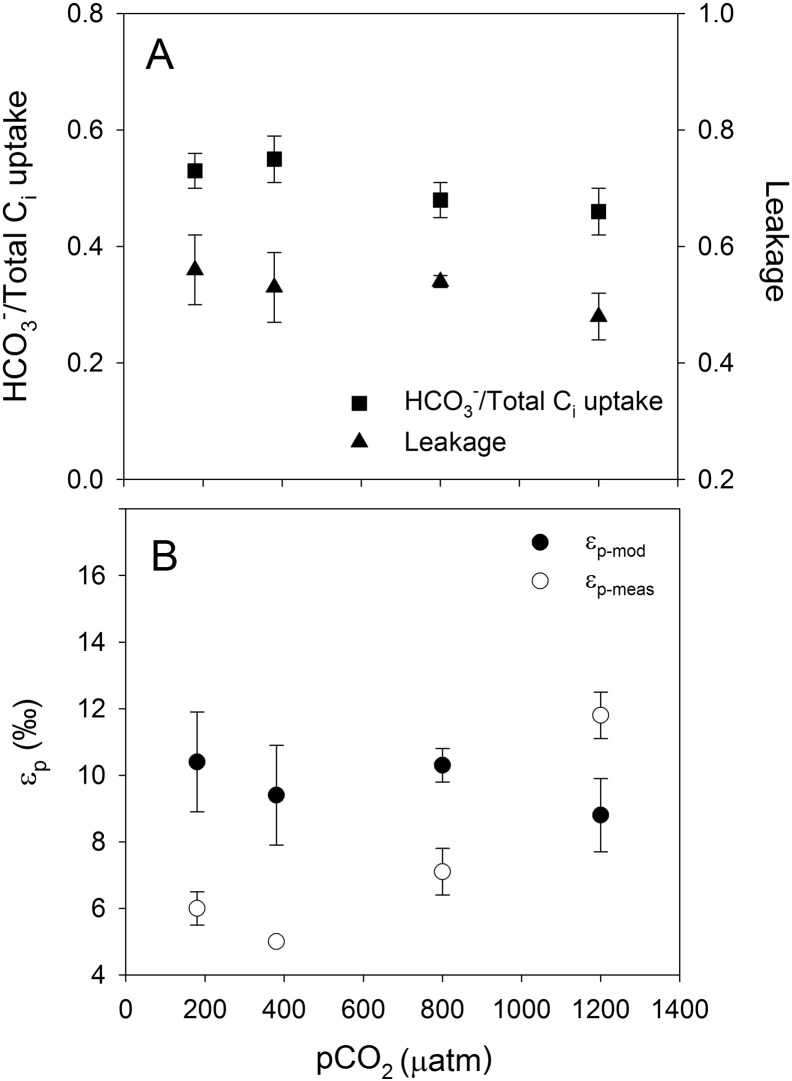
A) Relative HCO_3_^−^ contribution, leakage and B) ε_p-mod_ and ε_p-meas_ in *S. trochoidea*. Each data point represents the mean ± standard deviation (n = 3).

**Fig. 3:**
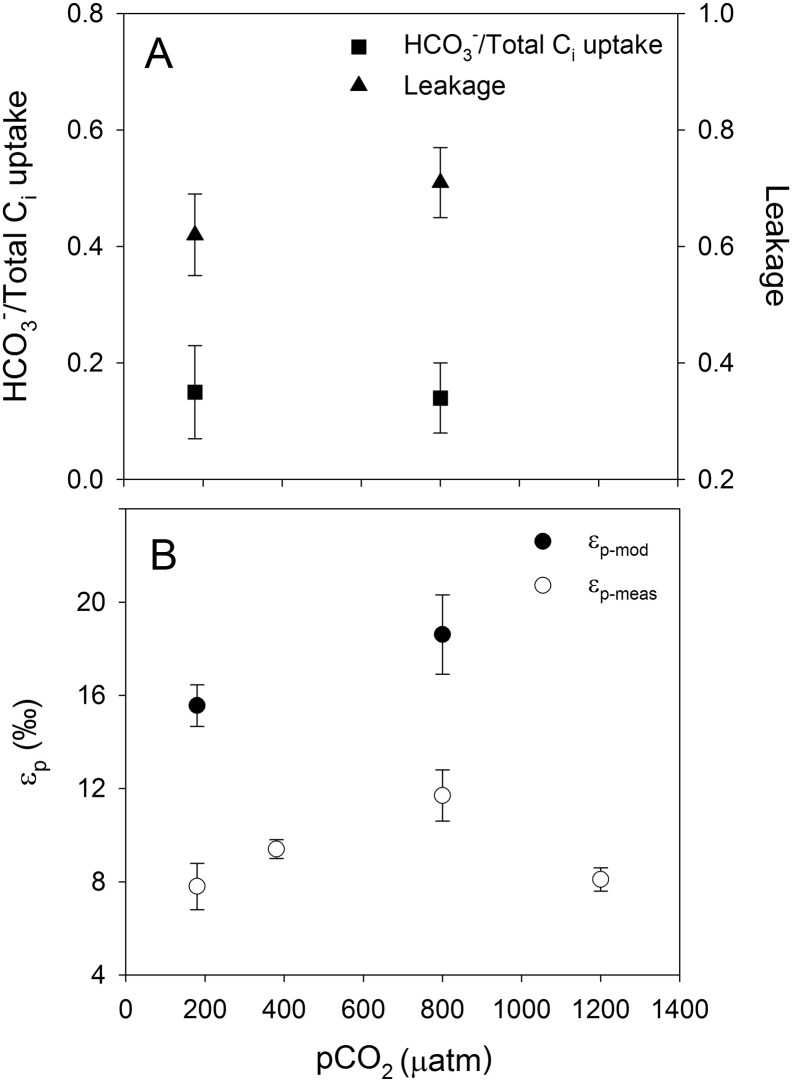
A) Relative HCO_3_^−^ contribution, leakage and B) ε_p-mod_ and ε_p-meas_ in *G. spinifera*. Each data point represents the mean ± standard deviation (n = 3).

**Fig. 4:**
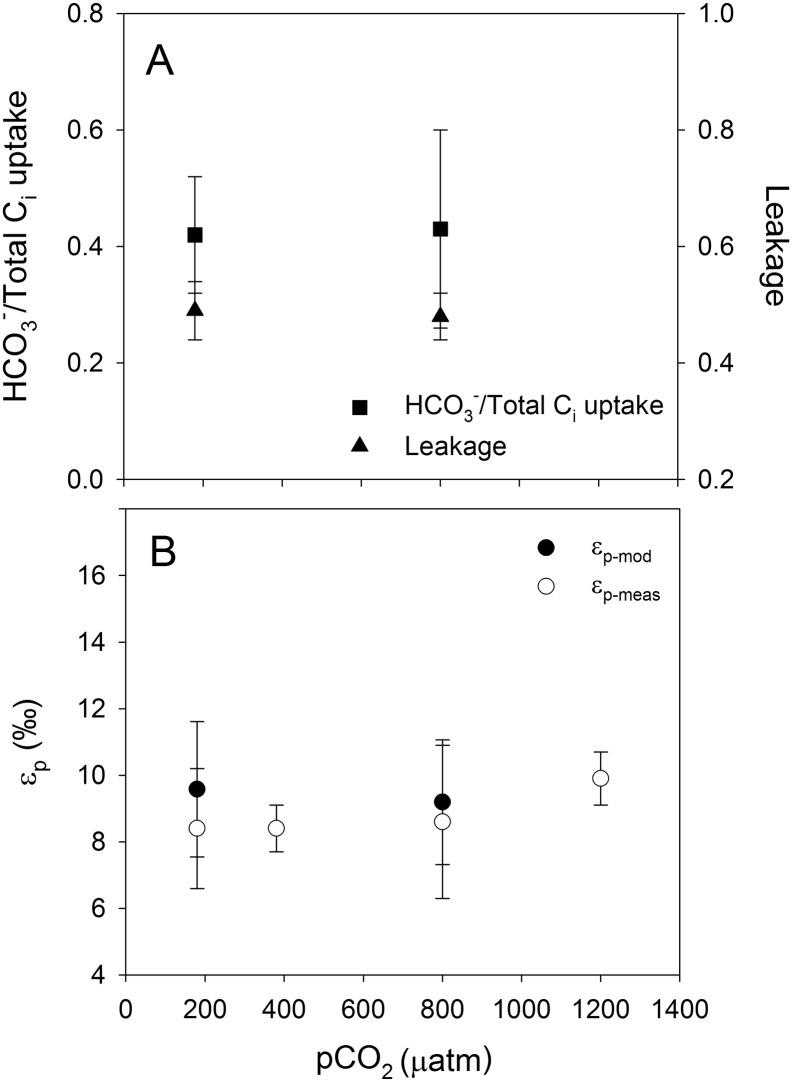
A) Relative HCO_3_^−^ contribution, leakage and B) ε_p-mod_ and ε_p-meas_ in *P. reticulatum*. Each data point represents the mean ± standard deviation (n = 3).

Relative HCO_3_^−^ contribution was around 0.2 in *A. fundyense* and *G. spinifera* (Figs. 1A and 3A; Table 1), whereas *S. trochoidea* and *P. reticulatum* showed higher values of ~ 0.5 (Figs. 2A and 4A; Table 1). In other words, in *A. fundyense* and *G. spinifera* approximately 80% of the C_i_ taken up is in the form of CO_2_, whereas in *S. trochoidea* and *P. reticulatum* this was 50%. There was a significant decrease in HCO_3_^−^ contribution with increasing CO_2_ concentration in *S. trochoidea*, while no such CO_2_-dependency was observed in any of the other tested species. Leakage differed significantly between the tested species, with values of up to 0.7 at 800 μatm *p*CO_2_ in *G. spinifera* (Fig. 3A; Table 1) and lowest average values of ~ 0.5 in *S. trochoidea* and *P. reticulatum*, respectively (Figs. 2A and 4A; Table 1). Only in *A. fundyense*, leakage was significantly CO_2_-dependent and increased from 0.44 to 0.63 (Fig. 1A; Table 1). For details on the kinetics of O_2_, CO_2_ and HCO_3_^−^ fluxes in *A. fundyense* and *S. trochoidea*, please refer to Eberlein et al. (2014).

### 3.3 C_i_ flux based ε_p_ calculations

Estimates for ε_p-mod_ are in the same range as ε_p-meas_ in *A. fundyense* and *P. reticulatum* (Figs. 1B and 4B; Table 1), while the model overestimated the fractionation by up to 5‰ and 8‰ in *S. trochoidea* and *G. spinifera*, respectively (Figs. 2B and 3B; Table 1). Except for *S. trochoidea*, ε_p-mod_ generally matches trends observed in ε_p-meas_. In *A. fundyense*, for instance, ε_p-mod_ increased significantly from 10.1 to 15.3‰, thereby closely matching ε_p-meas_ values (Fig. 1B; Table 1). Also in *G. spinifera*, ε_p-mod_ matches trends observed in ε_p-meas_, if the highest *p*CO_2_ treatment of ε_p-meas_ is excluded. In this treatment, carbon isotope fractionation dropped significantly, most likely due to 2.5-fold increased cellular carbon contents (see discussion in Hoins et al., 2015). In *S. trochoidea*, neither the relative HCO_3_^−^ contribution nor leakage showed a CO_2_-dependency; hence ε_p-mod_ did not match the increase in ε_p-meas_ with increasing CO_2_ concentration (Fig. 2B; Table 1).

## 4 Discussion

### 4.1 CA activity plays a minor role in C_i_ fluxes

By expressing CA, many marine algae species accelerate the otherwise slow interconversion between CO_2_ and HCO_3_^−^, thereby possibly facilitating the C_i_ uptake and internal C_i_ fluxes. In line with previous studies on dinoflagellates (Rost et al., 2006, Ratti et al., 2007), *A. fundyense* and *P. reticulatum* exhibit rather low eCA activities, even under low CO_2_ concentrations. In view of this, eCA is not expected to significantly influence C_i_ fluxes or ε_p_ in these species. In *S. trochoidea* and *G. spinifera*, however, eCA activities were high at all tested CO_2_ concentrations, comparable to values observed for temperate diatoms (Trimborn et al., 2009). Hence, the inhibition of eCA by the inhibitor DBS during the MIMS assay might have biased the C_i_ fluxes, i.e. underestimated CO_2_ uptake (Rost et al., 2003), thereby potentially causing an underestimation of ε_p_. As these were the species for which the model overestimated ε_p_ values, however, it can be concluded that eCA (and its inhibition by DBS during the C_i_ flux measurements) did not influence the C_i_ fluxes much.

### 4.2 Species-specific differences in C_i_ fluxes

The HCO_3_^−^ contribution differed considerably between the tested species. While *A. fundyense* and *G. spinifera* showed a strong preference for CO_2_, *S. trochoidea* and *P. reticulatum* used CO_2_ and HCO_3_^−^ in equal proportions. The latter contradicts with the findings of an endpoint pH-drift experiment, suggesting that *P. reticulatum* is not able to efficiently use HCO_3_^−^ (Ratti et al., 2007). Testing other dinoflagellates with a modified pH-drift method, including the genus *Protoceratium*, demonstrated that the high pH itself can affect growth and thus interpretations about the used C_i_ source based on pH-drift experiments must be considered with caution (Hansen et al., 2007). From an energetic point of view, high CO_2_ usage would be of advantage as CO_2_ can be taken up passively by diffusion, while HCO_3_^−^ is charged and thus has to be taken up by active uptake. And yet, the tested species covered a large part of their C_i_ demand by HCO_3_^−^, as observed in *S. trochoidea* and *P. reticulatum* (Figs. 2A and 4A).

Similarly high HCO_3_^−^ contributions were observed for other dinoflagellate species (Rost et al., 2006) and cyanobacteria (Price et al., 2008, Kranz et al., 2011). This preference for HCO_3_^−^ has been associated with the very low CO_2_-affinity of RubisCO type IB, which is expressed in cyanobacteria, and RubisCO type II expressed in dinoflagellates (Badger et al., 1998, Whitney and Andrews, 1998). To compensate for this low affinity, high amounts of C_i_ have to be accumulated, which in seawater can more easily be accomplished with the abundant HCO_3_^−^ ion rather than with CO_2_. In addition, HCO_3_^−^ is about 1000-fold less permeable to lipid membranes than CO_2_, making it the preferred C_i_ form to be accumulated within the cell (Price et al., 2008). In the case where species covered the majority of their C_i_ demand by CO_2_, as observed in *A. fundyense* and *G. spinifera* (Figs. 1A and 3A), one could therefore speculate about chloroplast-based C_i_ accumulation rather than pumping of HCO_3_^−^ at the cell wall (Badger et al., 1998). The observed differences in the preferred C_i_ source and likely consequences for internal C_i_ fluxes may also affect the overall leakage of cells.

Also leakage differed considerably among the species. *A. fundyense* showed a relatively low leakage of 0.44 at 180 μatm *p*CO_2_, which increased to 0.63 at 1200 μatm *p*CO_2_. Leakage in *G. spinifera* varied between 0.60 at 180 μatm *p*CO_2_ and 0.70 at 800 μatm *p*CO_2_. Leakage estimates in *S. trochoidea* and *P. reticulatum* were lower with ~ 0.50 and remained constant over the applied range of *p*CO_2_. These differences may be caused by different membrane permeabilities, which again potentially relate to the preferred C_i_ source. In fact, *A. fundyense* and *G. spinifera* both preferred CO_2_ over HCO_3_^−^ and likewise showed the highest degrees of leakage, thereby suggesting highly permeable membranes with respect to CO_2_. In these species, also CO_2_-related changes in the membrane permeability are indicated as they show significantly increased leakage under higher *p*CO_2_ (see also Eberlein et al., 2014 for *A. fundyense*, formerly *A. tamarense*).

### 4.3 Patterns in carbon isotope fractionation can be explained by C_i_ fluxes

Using results for HCO_3_^−^ contribution and leakage obtained in this study, carbon isotope fractionation was calculated and compared to previous measurements (Figs. 1B–4B; see also Hoins et al., 2015). Generally, there is a good agreement as ε_p-mod_ and ε_p-meas_ values were in the same range (*A. fundyense, S. trochoidea, P. reticulatum*) and/or followed the same trend (*A. fundyense, G. spinifera, P. reticulatum*; Figs. 1B-4B). Despite the overall agreement between flux-based estimates and directly measured carbon isotope fractionation, ε_p-mod_ was overestimated in *S. trochoidea* and *G. spinifera*. Such offsets could principally be attributed to biases in the C_i_ flux measurements, i.e. uncertainties in the estimation of HCO_3_^−^ contribution and/or leakage. It has been argued, however, that the MIMS approach tends to overestimate the HCO_3_^−^ contribution (due to the constant pH of 8.0 during the assay, see Burkhardt et al., 2001), and rather underestimates cellular leakage (due to fact that CO_2_ fixation does not cease instantly upon darkening, see Badger et al., 1994). Hence, by correcting for these potential biases, i.e. assuming slightly lower HCO_3_^−^ contribution and higher leakage values, we would actually overestimate the fractionation even more for *S. trochoidea* and *G. spinifera*.

An alternative explanation for the overestimation by the model may be attributed to the fractionation factor of RubisCO, which we assumed to be 28‰ (Raven and Johnston, 1991). Recent studies have found lower values, even as low as 11‰ as in the case of the coccolithophore *Emiliania huxleyi* (Boller et al., 2011). Even though there are no indications for such low fractionation values in the highly conserved type II RubisCO, a lower fractionation would bring modeled and measured ε_p_ values closer in *S. trochoidea* and *G. spinifera*. In *A. fundyense* and *P. reticulatum*, however, it would lead to underestimated ε_p-mod_. Hence, we would refrain from assuming much lower fractionation values for RubisCO type II in dinoflagellates in our calculations. Lastly, the fact that internal C_i_ fluxes were not taken into account might have also contributed to the offsets between ε_p-mod_ and ε_p-meas_. Models that incorporate internal C_i_ cycling have, however, caused even higher ε_p-mod_, as these processes work against the ^13^C accumulation within the chloroplasts (Cassar et al., 2006, Schulz et al., 2007) or the carboxysome (Eichner et al., 2015). Therefore, although the values and trends in carbon isotope fractionation are relatively well understood based on our physiological experiments, differences between theory and measurements are at present not fully resolved.

## 5 Conclusions

Our study demonstrates that carbon isotope fractionation in dinoflagellates can, to a large degree, be explained by considering their C_i_ fluxes. Relative HCO_3_^−^ contribution and/or leakage were CO_2_-dependent in *A. fundyense, S. trochoidea* and *G. spinifera*, which in turn can explain the CO_2_-dependency of their ε_p_ observed in previous studies (Hoins et al., 2015). To further advance our understanding of the ε_p_ patterns in dinoflagellates, C_i_ fluxes measurements should be performed at in situ pH (Kottmeier et al., 2014, Kottmeier et al., 2016) and ideally differentiate between ^13^C and ^12^C fluxes (McNevin et al., 2006).

## Figures and Tables

**Table 1:** Experimental conditions in dilute batch culture incubations (see also Eberlein et al., 2014, Hoins et al., 2015): average CO_2_ concentrations (μmol L^− 1^), total alkalinity (TA; μmol L^− 1^), dissolved inorganic carbon (DIC; μmol L^− 1^) and pH (NBS scale). HCO_3_^−^ contribution, leakage, modeled carbon isotope fractionation (ε_p-mod_) and measured carbon isotope fractionation (ε_p-meas_) was derived under the same conditions.

*p*CO_2_ μatm	CO_2_ μmol L^− 1^	TA μmol L^− 1^	DIC μmol L^− 1^	pH NBS	HCO_3_^−^ contribution	Leakage	ε_p-mod_ ‰	ε_p-meas_ ‰
*A. fundyense*								
180	5.9 ± 0.9^a^	2434 ± 3	1992 ± 10^a^	8.50 ± 0.06^a^	0.22 ± 0.03	0.44 ± 0.01^a^	10.1 ± 0.2^a^	9.0 ± 0.3^a^
380	11.5 ± 2.1^b^	2439 ± 1	2117 ± 8^b^	8.27 ± 0.07^b^	0.24 ± 0.04	0.46 ± 0.02^a^	10.6 ± 0.5^a^	10.2 ± 0.5^b^
800	25.9 ± 5.8^c^	2434 ± 2	2245 ± 8^c^	7.97 ± 0.10^c^	0.24 ± 0.04	0.53 ± 0.02^b^	12.6 ± 0.6^b^	12.7 ± 0.4^c^
1200	36.5 ± 9.3^d^	2418 ± 1	2283 ± 5^d^	7.83 ± 0.12^d^	0.23 ± 0.08	0.63 ± 0.05^c^	15.3 ± 0.8^c^	12.1 ± 0.2^c^

*S. trochoidea*								
180	6.6 ± 0.2^a^	2386 ± 1	1872 ± 2^a^	8.45 ± 0.01^a^	0.53 ± 0.03^a,b^	0.56 ± 0.06	10.4 ± 1.5	6.0 ± 0.5^a,b^
380	13.1 ± 0.5^b^	2388 ± 2	2096 ± 3^b^	8.21 ± 0.02^b^	0.55 ± 0.04^a^	0.53 ± 0.06	9.4 ± 1.5	5.0 ± 0.1^a^
800	28.8 ± 2.0^c^	2385 ± 1	2223 ± 3^c^	7.91 ± 0.03^c^	0.48 ± 0.03^b,c^	0.54 ± 0.01	10.3 ± 0.5	7.1 ± 0.7^b^
1200	41.5 ± 3.6^d^	2386 ± 4	2268 ± 9^d^	7.77 ± 0.04^d^	0.46 ± 0.04^c^	0.48 ± 0.04	8.8 ± 1.1	11.8 ± 0.7^c^

*G. spinifera*								
180	6.0 ± 1.1^a^	2447 ± 5	1962 ± 15^a^	8.50 ± 0.05^a^	0.19 ± 0.11	0.61 ± 0.01	15.6 ± 0.9^a^	7.8 ± 1.0^a^
380	11.7 ± 2.5^b^	2461 ± 12	2083 ± 1^b^	8.27 ± 0.07^b^	–	–	–	9.4 ± 0.4^a^
800	27.9 ± 7.4^c^	2475 ± 13	2224 ± 9^c^	7.96 ± 0.10^c^	0.19 ± 0.11	0.71 ± 0.01	18.6 ± 1.7^b^	11.7 ± 0.7^b^
1200	42.4 ± 7.9^d^	2459 ± 4	2293 ± 5^d^	7.78 ± 0.06^d^	–	–	–	8.1 ± 0.5^a^

*P. reticulatum*								
180	7.1 ± 0.5^a^	2460 ± 8	2002 ± 2^a^	8.43 ± 0.04^a^	0.44 ± 0.13	0.50 ± 0.06	9.58 ± 2.0	8.4 ± 1.8
380	13.9 ± 0.8^b^	2455 ± 2	2121 ± 4^b^	8.21 ± 0.02^b^	–	–	–	8.4 ± 0.7
800	31.0 ± 4.7^c^	2461 ± 12	2249 ± 23^c^	7.88 ± 0.08^c^	0.49 ± 0.19	0.48 ± 0.09	9.2 ± 1.9	8.6 ± 2.0
1200	45.2 ± 6.9^d^	2473 ± 19	2288 ± 16^d^	7.75 ± 0.05^d^	–	–	–	9.9 ± 0.8

Values represent the mean of triplicate incubations (n = 3; ± SD). Superscript letters indicate significant differences between *p*CO_2_ treatments (P < 0.05).
